# ACTIVATED SKN-1 ALTERS THE LONGEVITY TRAJECTORY OF LONG-LIVED C. ELEGANS MUTANTS

**DOI:** 10.1093/geroni/igae098.3630

**Published:** 2024-12-31

**Authors:** Chris Turner, Sean Curran

**Affiliations:** University of Southern California, Los Angeles, California, United States; University of Southern California, Los Angeles, California, United States

## Abstract

In the presence of stressful environments, the SKN-1 cytoprotective transcription factor is activated to induce the expression of gene targets that can restore homeostasis. However, constitutive activation of SKN-1 results in diminished health and a reduction of lifespan. Here we demonstrate the necessity to regulate the activity of SKN-1 for maintaining the longevity promoting responses associated with impaired daf-2/insulin receptor signaling, the eat-2 model of caloric restriction, and glp-1-dependent loss of germ cell proliferation. A hallmark of animals with constitutive SKN-1 activation is the age-dependent loss of somatic lipids and this phenotype is linked to the general reduction in survival in animals harboring the skn-1gf allele, but surprisingly, daf-2lf; skn-1gf double mutant animals do not redistribute somatic lipids which suggests the insulin signaling pathway functions downstream of SKN-1 in the maintenance of lipid distribution. As expected, eat-2lf; skn-1gf double mutant animals, which independently activate SKN-1, continue to display somatic lipid depletion in older ages with and without the skn-1gf activating mutation but animals lacking a proliferating germline do not redistribute somatic lipids, which supports a genetic model where SKN-1 activity is an important regulator of lipid mobilization in response to food availability to fuel the developing germline by engaging the daf-2/insulin receptor pathway.

